# Chess databases as a research vehicle in psychology: Modeling large data

**DOI:** 10.3758/s13428-016-0782-5

**Published:** 2016-09-01

**Authors:** Nemanja Vaci, Merim Bilalić

**Affiliations:** 1 Cognitive Psychology, Institute for Psychology, University of Klagenfurt, Universitätsstr. 65-67, 9020 Klagenfurt, Austria; 2 Department of Psychology, Northumbria University Newcastle, NE1 8ST, Newcastle Upon Tyne, UK

**Keywords:** Chess, Longitudinal dataset, Skill development, Expertise, Nonlinear analysis, Gender differences, Large datasets

## Abstract

The game of chess has often been used for psychological investigations, particularly in cognitive science. The clear-cut rules and well-defined environment of chess provide a model for investigations of basic cognitive processes, such as perception, memory, and problem solving, while the precise rating system for the measurement of skill has enabled investigations of individual differences and expertise-related effects. In the present study, we focus on another appealing feature of chess—namely, the large archive databases associated with the game. The German national chess database presented in this study represents a fruitful ground for the investigation of multiple longitudinal research questions, since it collects the data of over 130,000 players and spans over 25 years. The German chess database collects the data of all players, including hobby players, and all tournaments played. This results in a rich and complete collection of the skill, age, and activity of the whole population of chess players in Germany. The database therefore complements the commonly used expertise approach in cognitive science by opening up new possibilities for the investigation of multiple factors that underlie expertise and skill acquisition. Since large datasets are not common in psychology, their introduction also raises the question of optimal and efficient statistical analysis. We offer the database for download and illustrate how it can be used by providing concrete examples and a step-by-step tutorial using different statistical analyses on a range of topics, including skill development over the lifetime, birth cohort effects, effects of activity and inactivity on skill, and gender differences.

For a simple board game, chess has left a surprisingly big mark on scientific thought. Starting with mathematics, where chess has been used to formalize the concept of the game tree and its application in computer science (Zermelo, 1913), to the theory of emergence, describing how complex behaviors emerge from simple components (Hofstadter, 1979; Holland, 1998), and linguistics, where the combinatorial and rule-like properties of language have been illustrated with chess (Saussure, 1916), chess has been a building block of multiple scientific theories. However, nowhere has chess had such a great impact as in cognitive psychology. Chess is a deceptively simple game because it features clear-cut rules and a well-defined environment, which any child can learn easily. Yet, as anybody who has tried to play the game can testify, it is complex enough that some commentators have argued that there are more possibilities of game play in chess than there are atoms in the universe (Shannon, 1950). The mixture of the simple environment and rules, which enable experimental manipulations, and game complexity, which mimics the real world, have proven so appealing to many cognitive scientists that Nobel Prize winner Herbert Simon pronounced chess to be “the drosophila of cognitive science” (Chase & Simon, 1973).

Here we present another appealing feature of chess: its databases. Chess boasts highly organized and structured records of the activity of tens of thousands of people going back several decades. These databases present a gold mine for researchers interested in various topics in psychology. Similarly to those in other fields, chess databases offer the possibilities of applying data-mining and modeling approaches on large datasets and of investigating a number of cognitive effects (see Keuleers & Balota, 2015; Roring & Charness, 2007; Stafford & Dewar, 2013). Here we introduce one such database, the German database, and provide examples of how one can use this database to tackle topics such as skill development over the lifetime, birth cohort effects, effects of activity and inactivity on chess play, and even gender differences. Another goal of this article is to demonstrate how such a wealth of data can be analyzed appropriately. We therefore offer a detailed tutorial for using linear and nonlinear modeling approaches to investigate the above-mentioned topics.

Before describing the German database, we will briefly review the research in psychology that has employed the game of chess as its research domain. This overview will help us understand what kind of questions can be tackled with the archival approach of using chess databases. Unlike in many other domains, in chess it is possible to quantify the skill of practitioners through the use of the Elo rating system (Elo, 1978). The Elo rating is an interval scale with a theoretical mean of 1500 and a theoretical standard deviation of 200. Players compete against other rated players, and their ratings reflect their performance against these opponents. Elo rating offers a reliable and precise quantification of chess skill along the skill range. Beginners, for example, have ratings of around 800, novices around 1100, and average players around 1500, whereas expert players generally have ratings above 2000. The very best players, grandmasters, have ratings over 2500, and the ratings of top grandmasters these days go beyond 2800.

Cognitive psychologists have been using the rating feature in two ways. The first involves pitting two extreme skill groups, experts and novices, against each other to investigate cognitive processes. The use of the control novice group not only enables more statistical power for detecting the effects of interest (Campitelli & Speelman, 2013; Preacher, Rucker, MacCallum, & Nicewander, 2005), but also permits the drawing of conclusions about the nature of experts’ cognitive processes (Campitelli & Speelman, 2013; Kuhn, 1970; Wason, 1960). In that sense, the expertise approach (Bilalić, Langner, et al. 2010; Bilalić, Turella, Campitelli, Erb, & Grodd 2012), which enables the falsification of results obtained from experts through comparison with those from novices, is not dissimilar to the neuropsychological approach, in which “normal” participants were used as comparisons to patients (Shallice, 1988). This expertise approach has a long tradition (Chase & Simon, 1973; De Groot, 1978; Simon & Chase, 1973) and has been used to investigate memory (De Groot, Gobet, & Jongman, 1996; Gong, Ericsson, & Moxley, 2015), problem solving (Bilalić & McLeod, 2014; Connors, Burns, & Campitelli, 2011; Newell & Simon, 1972), decision making (Campitelli & Gobet, 2004; Moxley, Ericsson, Charness, & Krampe, 2012), pattern recognition (Chase & Simon, 1973; Gobet & Simon, 1996), and object recognition (Bilalić, Langner, et al., 2010; Charness, Reingold, Pomplun, & Stampe, 2001; Kiesel, Kunde, Pohl, Berner, & Hoffmann, 2009; Reingold, Charness, Schultetus, & Stampe, 2001). The results and theories deriving from this approach have been used as building blocks of computational models of cognition in general (Gobet & Simon, 2000; Gobet et al., 2001; Lane, Cheng, & Gobet, 2000; Richman, Staszewski, & Simon, 1995).

The other use of chess ratings exploits the full range of skill to quantify the effects of interest. This approach has been used to demonstrate the strength of the Einstellung (mental set) effect—that is, how much worse experts perform when the first solution that comes to their mind is a suboptimal one (Luchins, 1942). In this case, experts’ performance becomes similar to that of average practitioners, players three standard deviations below their nominal skill (for the mechanism behind this effect, see Bilalić et al. 2008a, 2008b, 2010). Similarly, when experts are taken out of their specialization areas, their performance becomes comparable to that of practitioners almost two standard deviations below their skill level (Bilalić, McLeod, & Gobet, 2009; Joseph & Patel, 1990; Voss, Tyler, & Yengo, 1983; for real-life consequences of this specialization effect, see Schraagen, 1993).

Not only the investigation of cognitive processes has profited from research on chess. The research on individual differences has often exploited the characteristics of chess to draw conclusions. For example, we know that children who do not take up chess as a hobby tend to be more agreeable than those who do (Bilalić et al. 2007b), which may explain the higher participation rates of men in chess, as they tend be less agreeable (Rubinstein, 2005). We also know that personality traits found in minority members tend to be opposite those found in groups that constitute the majority of practitioners, possibly because the minority group needs different traits to achieve success within the domain. Elite male chess players tend to be introverts, but the pattern is different for elite women players, who are rather extroverted (Vollstädt-Klein, Grimm, Kirsch, & Bilalić, 2010). In addition, we know that intelligence may play a role at the beginning of acquiring complex skills such as playing chess (Bilalić et al. 2007a), but that later other factors such as motivation and practice play a greater role (Campitelli & Gobet, 2011; Charness, Tuffiash, Krampe, Reingold, & Vasyukova, 2005; Ericsson, Krampe, & Tesch-Römer, 1993).

## Archival approach

The studies mentioned above investigated cognitive processes and individual differences by adopting the expertise approach—comparing experts and novices—or by employing the correlational approach of exploiting the presence of a reliable and precise rating system in chess. Recently, researchers have started to exploit the existence of a large amount of archival data for chess. Almost every national federation collects data about the chess players who compete in clubs and tournaments. Archives log players’ current rating, number of games played in a tournament, gender, age during a tournament, performance in the tournament, and changes in the ratings based on performance. The records provide a huge amount of data across the full range of age and expertise, which in turn enables researchers to investigate influences of age (Roring & Charness, 2007; Vaci, Gula, & Bilalić, 2015), gender differences (Bilalić, Smallbone, McLeod, & Gobet 2009; Chabris & Glickman, 2006; Howard, 2008, 2009; Knapp, 2010), skill acquisition trajectories (Gaschler, Progscha, Smallbone, Ram, & Bilalić, 2014; Howard, 2014b), and even the ongoing nature-versus-nurture debate (Bilalić, Smallbone, et al., 2009; Gobet, Campitelli, & Waters, 2002; Howard, 1999, 2001). The archival approach is not in contrast to the more experimental expertise approach previously described. Rather, one can consider the archival approach as complementary to the expertise approach, since it offers unique insight into processes over the whole lifespan and over the whole span of skill, with the advantage of dealing with a very large sample size. Here we provide access to such a database and illustrate how the wealth of these data can be used to tackle different psychological topics. Before describing the German database, we will briefly discuss a chess database that has previously been publicly offered (the FIDE international database; Howard, 2008).

## FIDE database

One of the oldest databases is the International Chess Federation (FIDE) database, which has been collecting the data of elite players since the introduction of the Elo rating system in the seventies. The FIDE database collects an imposing amount of information, including the rating scores for chess players at FIDE tournaments, numbers of games played per rating period, and the age and gender of players across the world (see Howard, 2006a). Since it is based on data collected over the past few decades, it enables researchers to investigate development over the course of a life. The FIDE database has been a useful tool in the past decade (see Howard, 2004, 2006b; Roring & Charness, 2007), which is unsurprising, given the wealth of data that it provides. Unfortunately, it suffers from a number of methodological problems (see Vaci, Gula, & Bilalić, 2014, 2015). One of the main problems is that for most of its time range, the FIDE database provides records only for the very best practitioners and excludes weaker players. The threshold for the inclusion of players in the FIDE database was historically set rather high, at 2200 Elo points, which includes only master-level players. The entrance threshold was moved to 2000 Elo points in the nineties, and it has been moved down several times since then, but only recently have records of all players, no matter how weak, been kept in the database. This threshold not only kept most players out of the FIDE database, but its constant lowering produced strong cohort and period effects, since the starting Elo scores for older players are much higher than those for the younger ones.

**Fig. 1:**
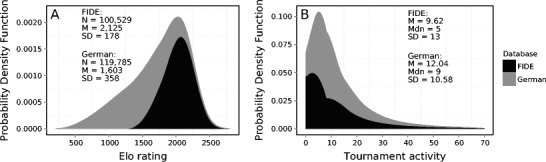
Probability density functions of chess skill (ratings) and activity (games per year in the FIDE [*dark gray*] and German [*light gray*] databases). (**a**) Probability density functions of chess skill. The datasets contain similar amounts of records, but they differ in the shapes of their distributions and coverage. The *y*-axis is the probability of rating scores across all players. (**b**) Probability density functions for activity, measured as the number of games played per year. The distributions of activity overlap, but the German database collects more records than the FIDE database does

The FIDE database also restricts logged tournament activity (Vaci et al., 2015). Tournaments are only recorded in the FIDE database if they have been registered as FIDE events, which comes with considerable costs that a good number of national federations cannot afford. Consequently, only a fraction of the games played by any player are captured. The difference between the FIDE database and the German database that we will present here is best appreciated if we consider the characteristics of those databases (see Fig. 1 and Vaci et al., 2015). For example, the FIDE database has multiple missing values for the number of games played per year (approximately 40 % of the database), which is not the case in the German database, which has approximately 2 % of missing values for the activity variable.

These restrictions in the skill range of players and their activity records could have serious consequences for the validity of conclusions from studies carried out using the FIDE database (see Vaci et al., 2014, 2015). Gender differences in skill are regularly found in the FIDE database (Howard, 2005, 2006b, 2014a; but see Bilalić & McLeod, 2006; Bilalić et al., 2007a), but they are mostly explained with reference to the gender participation disparities in other complete national databases (Bilalić, Smallbone, et al., 2009; Chabris & Glickman, 2006; see also the gender difference analyses in the Illustrative Examples section and the supplemental materials). In other words, it is impossible to estimate participation rates, because the FIDE database has strong skill restrictions. Our recent analyses (Vaci et al., 2014, 2015) have shown that the restrictions of the FIDE database also produce unreliable results when it comes to peak age (i.e., the age at which people have the best performance) and their declining trajectories after the peak. Similar research on the nature-versus-nurture debate (Howard, 2008, 2009) may also not stand closer inspection, since it is based on FIDE data that limit the estimation of practice, a variable that is essential for this particular investigation.

On the other side, the FIDE database provides other possibilities that are not covered by the German database. As we have already explained, the FIDE database collects records only from the best players. Therefore, it can be used together with the German database to investigate differences and changes in ratings for the very best practitioners. Because FIDE tournaments require certain fees from the organizers, often only the best players participate in those tournaments. However, the German and FIDE records overlap; that is, they collect records for a number of the same players. By using the biographical information of players, we calculated the overlap of the two datasets (see Vaci et al., 2015): Approximately 13,488 players are in both datasets. The more interesting difference between them is that the age of the players in FIDE differs from that in the German database (median ages of 39 vs. 33.5, respectively). Not surprisingly, players start to play FIDE tournaments later in life, probably when they reach a particular level of expertise, since the FIDE database required a high level of skill in the past. The information in the FIDE database and this difference can also give us information about the skill acquisition process, in that after reaching a particular level of expertise in national tournaments, players start participating in international ones.

Compared with the German database, the FIDE database is a truly international database, since all of the best players around the world have been included. Chassy and Gobet (2015) exploited this characteristic of the FIDE database and provided profound insight into the level of peacefulness (as indicated by the number of draws and the times at which those draws were made) across different cultures. Similarly, Gobet and Chassy (2008) used the FIDE database to investigate seasonal effects on the birth of the best players in the world, showing that most of the top players in the northern hemisphere were born in late winter and early spring. Finally, environmental changes (support on the national level, the introduction of chess software) cannot be studied with the German database, since this database collects records mostly from national players.

## German chess database

The German dataset of chess players collects records for a similar number of players (131,147 unique players with a total of 2,108,908 observations), but it is not plagued by the methodological limitations of the FIDE database. The German dataset of chess players represents one of the biggest national chess databases in the world, and it is arguably the best organized. Unlike the FIDE database, the German database collects records for all tournaments organized in Germany, including club championships that are not purely competitive. The database provided (see the supplemental materials) has records from 1980 to 2007, but the interested reader can find more recent records at the website of the German Chess Federation (www.schachbund.de). The website also describes how new data points can be compiled and downloaded.

Table 1 shows the variables collected in the German dataset of chess players. The ID variable identifies the individual players in the dataset. On the basis of this variable, we can see that the database contains the records of 131,147 players. This identification variable can be important when dealing with multilevel modeling, in which growth curves are adjusted for each player in the dataset. The Gender variable records whether the individual is a female or a male player. The database collects records for 7,789 female and 123,358 male players (approximately 6 % of players recorded are women). The Gender variable can be used to investigate the differences in rating scores and performance between the genders, but also to investigate possible reasons behind the strong differences in participation counts. The Country variable identifies the background and eligibility of the player: D indicates a German background (120,680 players); G, players with the same rights as domestic players (24); E, foreigners from Europe (1,480); A, foreigners from outside Europe (7,511); and S, players who are blocked from participation (95). The Birth and Year of Tournament variables code the birth year of the player and the tournament year. On the basis of these two variables, we calculated the ages of players for specific tournaments. In the case of the tutorial analysis, we used these variables to investigate birth cohort effects (see the supplemental materials), but also skill development functions. The next few variables record the performance of the players at the tournaments. For example, the variable Performance is calculated as the average DWZ (*Deutsche Wertungszahl*; see below) ratings of opponents plus the number of points gained, which is also measured in the dataset. The Games variable records the number of games played at a particular tournament (see Fig. 1 and the supplemental material for descriptive statistics and different ways to investigate the effect of games on rating scores). The Rating column lists the current DWZ points of players, and Expected Performance calculates the sum of all expected probabilities for a win or a draw (see the next section). In other words, for every individual game, the expected probability of a win or a draw changes for each player, and the expected performance is just a sum of all these probabilities. The Status of the player indicates whether this player is active or inactive, and the Stale variable indicates the difference in years between two consecutive tournaments for individual players.

**Table 1:** The sample of the values collected in the German dataset

Player	Gender	Country	Birth	Y_tour	Age	Per	Points	Games	Rating	Exp_P	Status	Games_Tour	Stale
73190.	*M*	D	1900	1992	92	0	2.5	7	1375	NA	Inactive	NA	0
73190.	*M*	D	1900	1993	93	0	1.5	6	1247	NA	Inactive	344	1
76220.	*M*	D	1900	1991	91	0	–1.0	0	1303	0.34	Inactive	0	0
76220.	*M*	D	1900	1992	92	1335	2.5	8	1313	0.55	Inactive	0	1
44188.	*M*	D	2000	2006	6	0	2.0	6	787	NA	Active	NA	0
44188.	*M*	D	2000	2007	7	681	2.0	6	774	2.66	Active	NA	1

Player, players’ unique identification; Gender, gender of the player; Country, national team or country of origin; Birth, year of birth; Y_tour, tournament year; Age, age of the player at the time of tournament; Per, players’ performance in the tournament; Points, observed points at the tournament; Games, number of games played at the tournament; Rating, current DWZ rating; ExpP, sum of players’ expected performance at the tournament; Status, active or inactive player; Games_Tour, total number of games played per tournament; Stale, number of years that passed between rated tournaments

A descriptive analysis of the German database shows that there is no restriction of the range of rating score values. The database collects data for all players, starting from beginners and extending all the way to the best players in the world (see Table 2 and the Age Effects subsection below). The initial Ingo rating system was changed at the beginning of the 1990s to the current German evaluation number (DWZ), resulting in the transformation of all scores in the dataset. The new DWZ system is based on the same assumptions as the Elo rating, resulting in a strong correlation between estimated scores of .93 (see Bilalić, Smallbone, et al., 2009). In the Appendix, we provide an analysis and simulation that confirm that the Elo and DWZ rating systems are essentially the same. It is important to note that the system of data collection has not been changed since the beginning and has always included all registered players and tournaments. Therefore, the birth cohort and period effects are small and nonsignificant (see the Birth Cohort Effects subsection below), and the number of games per rating period has been more accurately recorded, which is essential for estimation of the importance of practice for cognitive processes (see the (In)activity Effects subsection).

**Table 2:** Descriptive statistics for the variables in the dataset

	Mean	Median	*SD*	Min	Max
Birth	1963	1964	18.7	1900	2001
Tour_year	2000	2000	4.2	1981	2007
Age	37	35	18.1	5	95
Games	5.6	6	3.03	0	91
Rating	1592	1628	369.1	1	2813
Performance	1145	1466	813	0	3931
Expected performance	2.8	2.7	1.8	0	63.8
Points	2.7	2.5	2	–1	39

Birth, year of the player’s birth; Tour_year, tournament year; Age, age of the player; Games, number of games played at the tournament; Rating, current DWZ rating at the tournament; Performance, performance of the player at the tournament; Expected performance, sum of expected performance for each individual game at the tournament; Points, number of points each player scored at the tournament

As we can see in Table 2, the database collects records for players born between 1900 and 2001; thus, it contains records for all age ranges of players (from younger ones to octogenarians). However, the Tournament variable tells us that most of these records come from tournaments organized in the 2000s. The number of games per tournament, and the expected and obtained points, are right-skewed—that is, most values are small numbers, and the distribution has a long right tail. Contrary to this, the ratings of players are normally distributed, with a mean of 1592 and a standard deviation of 369. Overall, we can see that most records are well-represented in the database and can be used to examine different research questions. Additionally, the analysis can be performed on the level of potential population (including all players), but also on the level of chess masters (including only top-performing players). However, the differences in the distributions of different variables imply that one should be careful when analyzing and modeling the data.

## Illustrative examples

The German database can be used in different ways, but here we provide practical analyses on the topics of skill development over the years, birth cohort effects, the influence of expertise-related activity and inactivity on skill, and the gender differences. Because of space constraints, all R codes and estimated coefficients from the analysis can be found in the supplemental materials, where the German database can also be downloaded (https://osf.io/4zce8/). Moreover, in the supplemental materials we describe how skill development over the years can be analyzed with a step-by-step approach, which starts from linear models, moves to linear mixed-effect modeling and polynomial models, and finally includes nonlinear models (generalized additive mixed models). Every analysis is followed by inspection of the model, interpretation of the effects, and critique of the model, and at the end we provide practical advice about this type of modeling. Here we present the main results from the models, together with their interpretation.

### Age effects

**Fig. 2:**
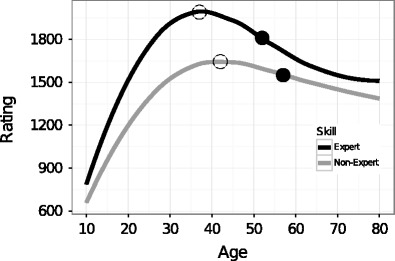
Estimated age-related functions for expert (*dark gray*) and nonexpert (*light gray*) players. The points on the function represent the first and second derivatives of the function. The first point (*white*) is the maximum of the function, or peak value for chess players. The second point (*black*) is the stabilization of the decline with age. Both the maximum and stabilization points are observed earlier in the case of experts

Chess has often been used to study expertise and the development of skill across the lifespan. Most of the studies in the domain have focused on the “age is kinder to the initially more able” hypothesis, which postulates that more-able people (experts) decline less over the years than do their less-able peers (nonexperts). For example, Roring and Charness (2007; see also Almuhtadi 2011) used the FIDE database of chess players to investigate the difference between the age-related declines of expert and nonexpert chess players. They observed that chess experts experience a smaller decline in later years than do nonexpert players. We used linear mixed-effect modeling (Baayen, Davidson, & Bates 2008; Bates, 2005; Bates, Maechler, Bolker, & Walker 2015; Fang, 2011; Gelman, Carlin, Stern, & Rubin, 2014; Gelman & Hill, 2007; Kuznetsova, Brockhoff, & Christensen, 2013; Pinheiro & Bates, 2006) on the German database of chess players and showed that the “age is kinder to the initially more able” hypothesis should be updated to take into account the tail of the age-related function (Vaci, Gula, & Bilalić, 2015; see the section Is Age Kinder to the Initially More Able? in the supplemental materials for coefficient estimations). In particular, experts stabilize their decline in later years. On the one hand, the decline of experts is proportional to their increase to the peak, making the decline more pronounced than that of lesser players. On the other, the postpeak decline starts to stabilize after a certain point, and the point at which experts’ decline starts to stabilize occurs sooner than among nonexperts (see Fig. 2). Age may be crueler to experts when we compare immediate declines from the pinnacle; however, their accumulated knowledge obtained though practice helps experts to preserve their skill as they get older.

### Birth cohort effects

It should be kept in mind, with reference to chess datasets, that the observations are not completely longitudinal. Most people play during one period of their lives after which they stop completely, or they may resume playing later in life. Additionally, the logistical procedures behind the data collection have changed over the years. For example, the threshold for including chess players in FIDE has decreased from 2200 to 1500 Elo points. This usually results in a strong correlation between birth cohorts, age, and time periods (Fooken, 1990; Glenn, 1976). An additional challenge is that society and technology are rapidly changing as more materials for studying and practicing chess become available. This may result in a faster increase of underrated younger players, who take more points from older chess players. Regardless of how birth cohort data are examined, these effects may be confounded with one another (Glenn, 1976; Mason, Mason, Winsborough, & Poole, 1973). That is, age trends can be influenced by changes in logistical procedures (period effects) and by changes in society (birth cohort effects). Therefore, it is of interest to examine whether these effects remain strong in the case of the German database.

In the case of chess datasets, there are no data for the whole lifespans of players. In the German database, data are collected for players born between the years 1900 and 2001, and for tournaments played from 1981 until 2007. This results in the data collected ranging from approximately 1 to 25 years of play for different players (*M* = 6.9, *SD* = 5.1). Here, we investigated whether the aging function changes in different birth cohorts by dividing players into three groups: (1) players born after 1980, (2) players born between 1940 and 1980, and (3) players born before 1940. This resulted in 39,077 players between 5 and 27 years of age (*M* = 15.2, *SD* = 3.4) in the first group, 74,811 individuals between 10 and 67 years of age (*M* = 37.7, *SD* = 11.8) in the second group, and 17,259 individuals between 46 and 95 years of age (*M* = 66.9, *SD* = 6.4) in the third group.

**Fig. 3:**
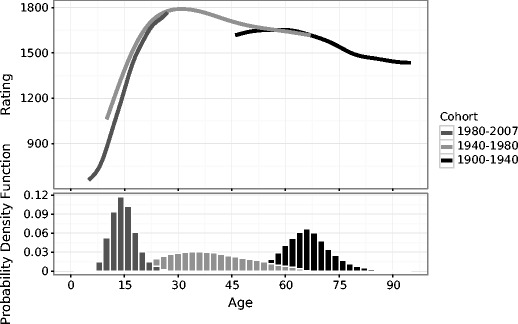
Birth cohort differences across the age-related skill function. The upper graph shows changes of rating scores (*y*-axis) over different ages (*x*-axis) for three different birth cohorts (color groups). The first group are all players born in the period 1980–2007 (*dark gray*), the second group are players born in the period 1940–1980 (*light gray*), and the third group are players born in the period 1900–1940 (*black*). The lower graph shows the probability density distributions of age separately for the three birth groups, where the *y*-axis shows probability density function values—that is, the relative likelihood for the age variable to take on a given value. The probability density functions show that the players in the second (*light gray*) and third (*black*) groups have wider spreads on the age variable than do the players in the first group (*dark gray*)

We used generalized additive models (GAMs) to fit a nonlinear regression for the skill function over the age of chess players (Hastie & Tibshirani, 1990; Marx & Eilers, 1998; van Rij, Hollebrandse, & Hendriks, in press; Wood, 2006). GAMs use spline smoothing over the rating scores, capturing every nonlinear trend in the data (for more details, see the Generalized Additive Mixed Modeling section in the supplemental materials). In this way, we get a function that is most truthful to the real trends in the raw data. The results presented in Fig. 3 show that birth year does not confound the skill function across the age groups—the three cohorts align with each other almost perfectly (see the Birth Cohort Effects section in the supplemental materials for coefficient estimations). There are small differences in the tails of the functions, but this is expected, since the tails of the functions contain fewer players on which to base the estimates (see the lower graph for probability density functions, the relative likelihoods for the variable to take on given values).

We also investigated whether younger players have a stronger increase of rating scores at the beginnings of their careers than do older players. The older group consisted of all players born between 1970 and 1985, and the younger group was made up of people born after 1985. The linear mixed-effect regression (Baayen et al., 2008; Baayen & Milin, 2010; Bates, 2005; Radanović & Vaci, 2013) was fitted to the increases of the function before the peak for young and old players using the lme4 package in R (Bates et al., 2015; R Development Core Team, 2013).

**Fig. 4:**
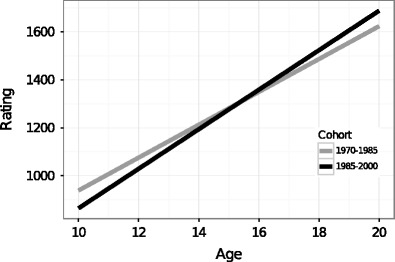
Increases before the peak for two birth cohorts. The first group are older players (*light gray*) born between 1970 and 1985, whereas the second group are younger players (*black*) born between 1985 and 2000

The results show that the increases before the peak differ between older and younger players in the dataset (see Fig. 4). Younger players in the dataset start with lower ratings and have a steeper increase to the peak, whereas older players start with a higher rating and increase more slowly. This effect can be interpreted as an increased number of young, inexperienced players enrolling in competition, and therefore lowering the starting ratings. Due to the increased availability of chess materials in the last few decades, young players also tend to develop faster than players from previous decades. However, this result may also indicate a possible trade-off between initial skill and increase of this skill. In our previous study, we showed on the individual level that chess players who have stronger starting positions experience a shallower increase to the peak, whereas players who have a weaker rating increase at a higher rate (see Vaci et al., 2015).

### (In)activity effects

Previous studies in the domain of expertise have proposed that one needs to be immersed in a domain for about 10 years to become an expert, the so called “10-year rule” (Ericsson et al., 1993; Ericsson & Charness, 1994; Simon & Chase, 1973; Simon & Gilmartin, 1973). Currently there are debates about what kind of activity leads to improvement of performance (Baker, Côté, & Abernethy 2003; Campitelli & Gobet, 2008; Charness et al., 2005; Ericsson et al., 1993; Gobet & Campitelli, 2007; Hambrick et al., 2014; Sloboda, Davidson, Howe, & Moore, 1996), but hardly anyone disputes the fact that activity is necessary to acquire skill. The German database collects records for tournament activity—that is, games played, which can be defined as expertise-related activity (Vaci et al., 2015). To get a better picture of the influence of activity on expertise, we investigated how both activity and inactivity affect the rating scores. We calculated the time difference between logged tournaments for each player, the measure we call “stale play,” because it illustrates the inactivity time span. We again fitted GAMs on the rating scores of the players using tensor^1^ interactions of the age of players and activity (number of games played for each player in 1 year). Additionally, we investigated the change of rating scores for each player across the values of stale play, which is the inactivity measure [see the (In)activity Effects section in the supplemental materials for coefficient estimations].

**Fig. 5:**
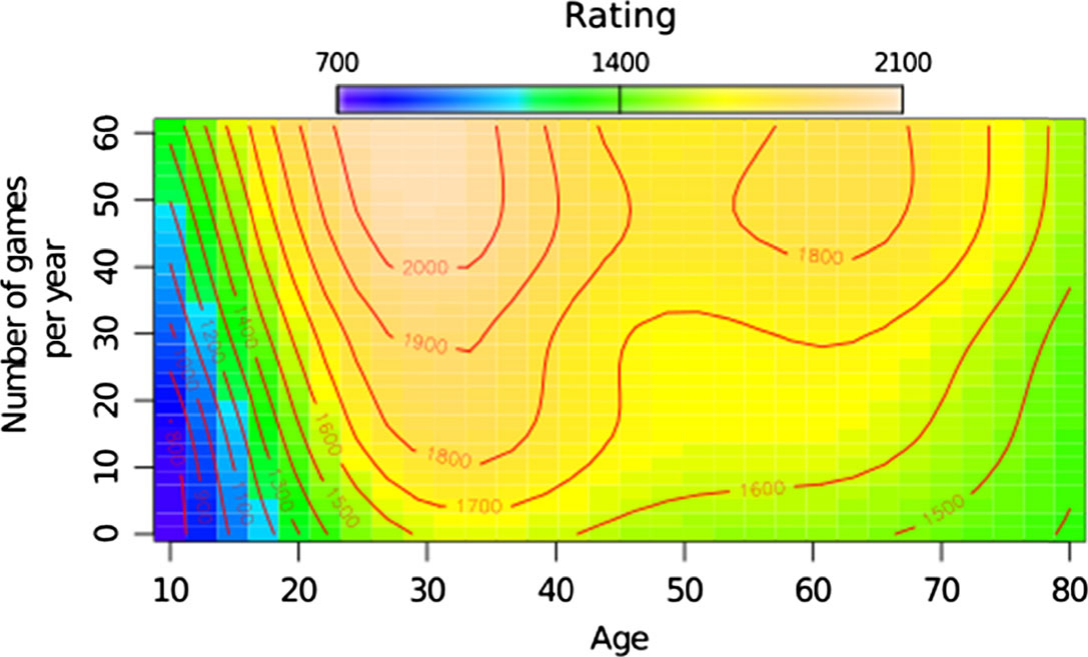
Interaction of age and tournament play for rating scores. The ages of players are presented on the *x*-axis, and tournament activity is presented on the *y*-axis. The colors in the graph present changes of rating scores: Darker colors are areas with lower rating scores (approximately a 700 rating), whereas brighter colors represent increases of DWZ scores (up to 2100 rating points). The contour lines (*red*) in the graph show areas along which players have constant rating scores

The results show that activity changes the age-related function: More activity results in smaller declines of rating scores, and vice versa. In Fig. 5, the *x*-axis indicates the ages of players, the *y*-axis shows their activity in tournaments, and the colors in the graph indicate changes of DWZ ratings, with darker colors representing lower DWZ scores and brighter colors representing higher scores. Additionally, the red lines in the graph (contour line or isoline) indicate a curve along which the function has a constant value. When these lines are closer together, the magnitude of the change is larger. In this case, they represent areas with the same rating. The influence of the players’ ages on the changes of ratings is evident from the lower half of the graph, when we follow contour lines over the ages of players. The players start with a DWZ rating around 800 points when they are 10 years old; around the age of 20 they increase to 1300 points, after which their scores improve to approximately 1600 points at around 30 years old. In their 40s a slow decline of DWZ points begins, which stabilizes around 70 years old with less than 1500 points. However, the effect of tournament activity changes this development, which is observed in the upper half of the graph. In the case of younger players (until their 20s), playing more games increases performance rapidly: We can see a steep increase of ratings from 800 to 1300. This is also evident for players across all ages, but with a shallower increase. Interestingly, the analysis shows a possible expertise window that is related to both age and practice. The players in their early 30s who play approximately 40 games per year have more than 2000 DWZ points. This expertise window covers players from their late 20s until the end of their 30s and strongly depends on activity. After their 30s, DWZ rating scores decline from 1800 to 1600 for players. Importantly, if the once-declining players increase their play from 10 to 30 games per year, this decline slows down, illustrating the preserving effects of immediate activity, shown as a contour line that rises with activity around 40 years of age.

**Fig. 6:**
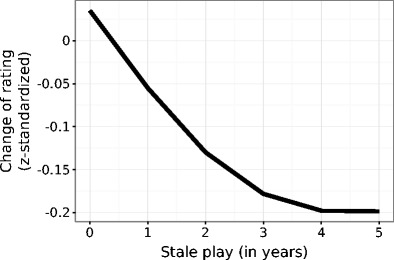
Effect of inactivity in play on declines of rating scores. The *y*-axis shows changes of standardized ratings for every player, whereas the *x*-axis shows inactivity, or the time between rated tournaments in years

Results for the effect of inactivity show that the longer the time span between tournament games, the more that players decline later (see Fig. 6). This decline follows a negative logarithmic function—that is, inactivity is strong at the beginning, taking many points from players. However, the decline of rating scores due to inactivity becomes stable; thus, the effect of being inactive for 5 years is no different from that of being inactive for 4 years.

### Gender differences

Gender differences could also be investigated using the database. We included the Gender factor in the previous GAM analysis (see the Gender Differences section in the supplemental materials). In this way, we adjusted the previously modeled tensor interaction between age and games on rating scores for each level of the gender variable. As we mentioned previously, there are more male than female players (only 6.3 % of the database are female players). However, both genders are well represented in the database, since there are 123,358 male and 7,789 female players. The results from our GAM models show that the interaction effect between age and activity is less wiggly in the case of female players (edf_female_ = 21.6, edf_male_ = 23.52).

**Fig. 7:**
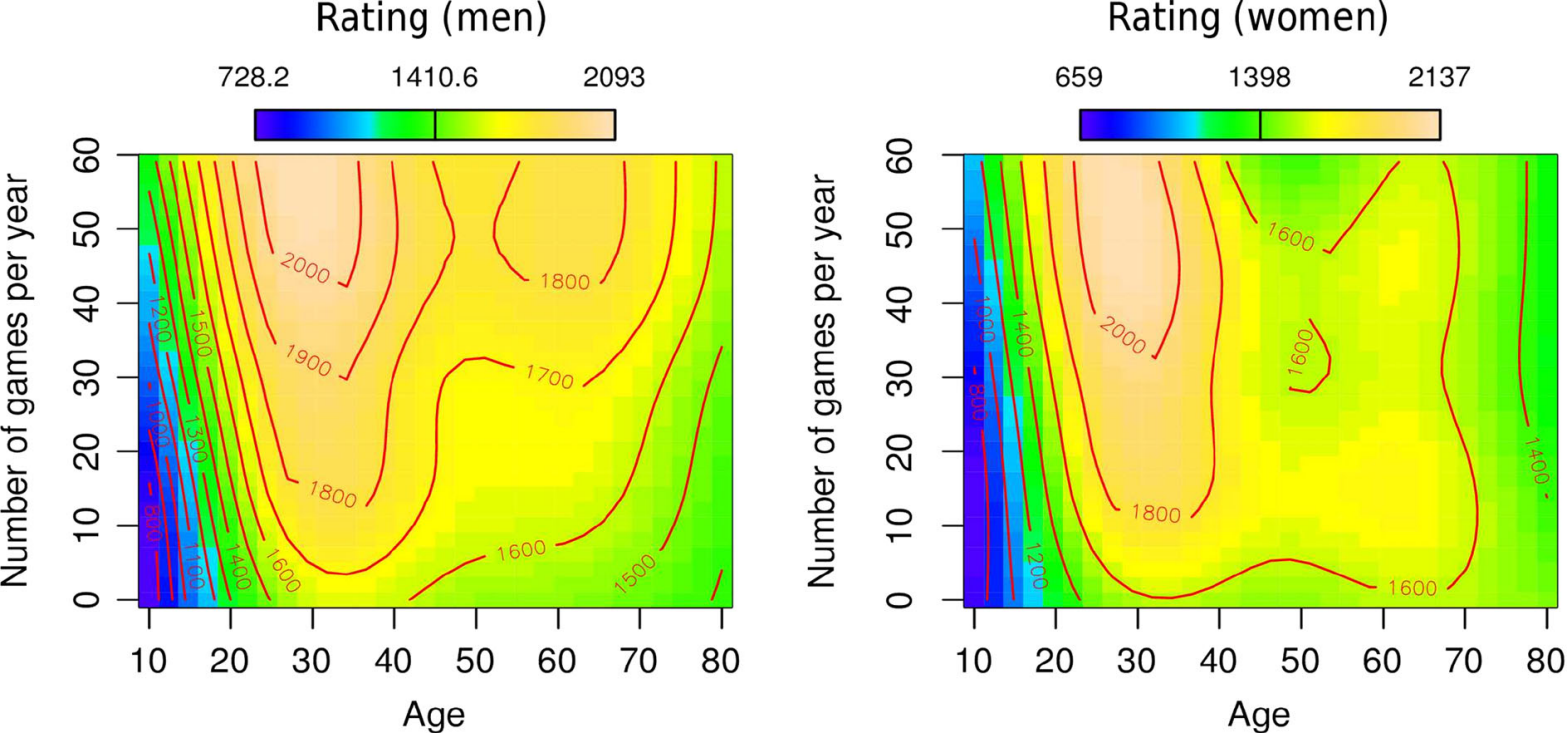
Interaction of age and tournament play on rating scores for men (*left*) and women (*right*). The ages of players are presented on the *x*-axis, and tournament activity is presented on the *y*-axis. The colors in the graph present changes of rating scores, with darker colors showing areas with lower rating scores (approximately a 700 rating), and brighter colors representing increases in Elo scores (up to 2100 rating points)

The effects of age and activity for different genders can be interpreted from Fig. 7 (men on the left side, women on the right). The *x*-axis represents the ages of players, the *y*-axis shows activity in tournaments, and the colors in the graph indicate changes of ratings, with darker colors representing lower DWZ scores and brighter colors representing higher scores. As in the previous case, the red lines indicate areas where the function has constant values. The male players start with a rating of around 700 points when they are 10 years old, as compared with women, who start with approximately 650 points. Around the age of 20, both groups increase to 1300 points, after which their scores improve to approximately 1600 points by around 30 years. This increase is more pronounced for men. Beginning in their 40s,a slow decline of DWZ points stabilizes around 70 years with less than 1500 Elo points.

The effect of tournament activity changes this development, which is observed in the upper half of the graph. Both women and men increase considerably in skill when they are young; thus, at this age the skill acquisition period does not change between genders. In contrast, the previously identified window of expertise, which occurs between the end of the 20s and the late 30s, changes between genders. Women need a smaller amount of activity (number of played games per year) to reach this window. Compared with men, who need around 43 games played per year to reach DWZ ratings of 2000, women need approximately 33 games per year. After their 30s, there is a decline of rating scores for all players. But the previously identified preserving effects of immediate activity—that is, when players increase the number of games they play per year—cannot be identified in the case of women. In other words, female players observe a stronger decline of rating scores later in life. Overall, the differences between genders are not that strong in the case of the German database, at least not in the case of this analysis.

## Conclusion

The German database offers one of the best longitudinal datasets available for use in psychological research. It does not suffer from the methodological problems inherent in other publicly available databases, such as FIDE (Howard, 2008). It opens new possibilities for the investigation of the multiple factors underlying expertise and skill acquisition with an archival approach. On the other hand, the social factors behind chess performance can also be measured and extracted from this type of dataset. For example, researchers can examine dropout rates and the factors that influence players to stop participating in tournaments. Information about players can also be used to investigate topics such as gender differences.

We believe that the German database provided here is a clear improvement on the previously employed FIDE dataset. That said, it is important to note some restrictions of the German database that may not be present for the FIDE database. The German database is restricted to players from Germany, unlike the FIDE database, which includes international players. This prevents intercultural studies, such as the clever study carried out by Chassy and Gobet (2015), who used the FIDE database to investigate cultural differences in risk taking across different countries and religions (see also Gobet & Chassy, 2008). The FIDE database suffers from a number of methodological problems, and therefore may not be of much use for tackling a number of the topics we described above. However, its international scope makes it suitable for investigating cultural differences. In other words, both databases have their advantages, and the database choice does not depend on general preference, but rather on which database can provide better resources for answering the question at hand.

The archival approach described here has its positive and negative sides, when compared to the experimental approach. In the case of the chess datasets, one loses the possibility to experimentally control for factors behind skill development or other topics that we have illustrated in the article. In the experimental approach, researchers try to control potential confounds in advance (before running the experiment). This is not possible in the case of an archival approach, in which potential data confounds already reside in the data. On the positive side, the number of observations collected in the database eliminates doubts about low statistical power (Maxwell, 2004). Psychologists usually deal with multidimensional problems, in which multiple factors interact to influence the process of interest. The number of different measures and the number of observations in the archival approach provide us with the possibility to model data and see how these factors interact, in contrast with the experimental approach, in which one would need numerous experiments to achieve this ability. In other words, the archival approach may not only be more efficient, but also may be more ecologically valid than the experimental approach.

Researchers can also perform their analyses in various ways because of the large sample size. Previous studies have shown that bootstrapping and “slicing” the dataset—that is, identifying individuals with certain conditions and comparing effects within the group—could be an effective way to analyze these data (see Stafford & Dewar, 2013). In our previous study, we showed that multilevel modeling and cross-validations can be used to make effective models (see Vaci et al., 2015). Finally, here we showed how nonlinear regression analysis and data exploration methods could be used to investigate theoretical and data-driven effects (see also Keuleers & Balota, 2015).

The German database offers one of the best longitudinal datasets available for use in psychological research. It complements the currently prevailing experimental expertise approach by opening up new possibilities for the investigation of the multiple factors underlying expertise and skill acquisition with the archival approach. Our hope is that by offering the database for download and providing practical examples of possible analyses (together with R codes for the analyses and a step-by-step tutorial), we will entice researchers to use these data, which may give answers to many questions that one could not answer with other available data.

## Figures and Tables

**Fig. A1:**
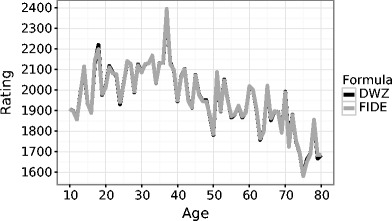
Comparison of the two methods for calculating chess ratings: German evaluation number (DWZ, *black*) and the Elo method (*gray*)

## Appendix: DWZ versus Elo ratings

Chess skill is measured on a continuous (interval) scale, which reflects the performance of players against other players. The most common measure is the Elo rating, named after the mathematician Arpad Elo, who introduced this type of measurement in chess (Elo, 1978). The Elo rating is inferred from paired comparisons—that is, player-versus-player outcomes. The Elo rating increases or decreases on the basis of game outcomes; in particular, after every game, the winning player takes points from the losing player. The formula used to update the ratings in the case of the international database (FIDE) is *R*_*a*_^′^ = *R*_*a*_ + *K*(*S*_*a*_ − *E*_*a*_), where *R*_*a*_ is the current score, *K* is the adjustment factor for the sensitivity of change, and *S*_*a*_ and *E*_*a*_ are the observed and expected scores, respectively. Compared with the FIDE database, the German database calculates performance using the Deutsche Wertungszahl (“German evaluation number”), or DWZ. The DWZ is calculated in the following way:

$$ {R}_a^{\prime }={R}_a+\frac{800}{K+n}\ast \left({S}_a-{E}_a\right). $$

Both datasets calculate the observed and expected outcomes in the same manner. In the case of the expected outcomes, both datasets use the following formula:

$$ {E}_a=\frac{1}{1+{10}^{\left({R}_b-{R}_a\right)/400}}, $$

where *R*_*a*_ and *R*_*b*_ represent the rating points of two players. The expected score is calculated for each player in the paired match and represents the probability of obtaining a win or a draw. In cases in which a player’s observed tournament performance exceeds his or her expected scores, the Elo rating is adjusted upward, and vice versa. In the case of observed outcomes, a win is coded as 1, a draw as .5, and a loss as 0 points.

The main difference between the formulas is in the calculation of the adjustment or development factor (*K* factor). The *K* factor is set up as three possible values in the FIDE database: (1) *K* = 40 for a new player who has a rating below 2300 and fewer than 30 games played, or a player younger than 18 years old; (2) *K* = 20 for players with a rating below 2400 Elo points; and (3) *K* = 10 for players with at least 2400 Elo points and at least 30 games played. The *K* factor in the case of the DWZ rating consists of the fundamental value *E*_0_, the acceleration factor *a*, and the braking value *B*, and follows the formula *K* = *a* * *E*_*o*_ + *B*. The fundamental value is calculated with

$$ {E}_o={\left(\frac{R_a}{1000}\right)}^4+J $$

where *J* depends on the age of the player (*J* = 5 for players up to 20 years old, *J* = 10 for players between 21 and 25, and *J* = 15 for players above 25). The acceleration factor (*a*) helps younger players improve faster, and it is calculated

$$ a=\frac{R_a}{2000} $$

only if the player is less than 20 years old and has achieved more points than expected; otherwise, this value is set to 1. Finally, the braking value (*B*) adjusts the decrease of weak players and is calculated $B=e\frac{1300-{R}_a}{150}-1$ for players with a rating under 1300 and who achieve less than or equal to their expected points. One would think that a difference in the calculation of ratings should also result in different rating scores, rendering the rating systems not comparable. However, here we show that this is not the case, and that both rating systems give essentially the same outcomes.

To investigate whether the outcomes of the two rating systems are comparable, we simulated three different datasets. In the first dataset, we simulated 100 paired matches between players with different rating scores; that is, we randomly sampled 2,000 values in the range from 800 to 2800 that represented the Elo ratings for 2,000 different players. In the second step, we randomly assigned these players into pairs and assumed that each pair was playing only one game in that rating period, in which one of them has to win—the possibility of a draw being excluded. We kept the *K*, *J*, and *a* values constant to investigate possible different outcomes from the main part of the formulas. In the third step, we simulated random outcomes that occurred in these chess matches, assigning 1 point for a win and 0 points for a loss. Finally, we updated the rating scores using both formulas, FIDE and DWZ, taking into account the expected and observed outcomes in the simulated matches. The results showed that the correlation between the updated ratings calculated with the international chess system and the DWZ system is .999. The ratings calculated with the two systems presented only small differences (*M* = 5.10, *SD* = 5.74, Min = 0.0007, Max = 21.67).

In the second dataset, we repeated the procedure used in the first one. Importantly, besides simulating the rating scores for 2,000 different players, we also simulated their hypothetical ages, from 10 to 80 years. This additional variable enabled adjustment of the *K*, *J*, and *a* values for every paired match. As in the previous case, we randomized the outcomes of the matches and updated the rating scores for both players in each paired match. We obtained a pattern similar to the one in the first simulation, with a correlation of .999, but with larger differences in the updated ratings (*M* = 9.20, *SD* = 13.15, Min = 0.0002, Max = 113.26).

Finally, in the third dataset we simulated the rating scores for four different players, assuming that they were playing throughout their lifetimes (starting at 10 and finishing at 80 years). The rating scores over the age range were simulated by using coefficients from the linear mixed-effect model fitted for the Vaci et al. (2015) study. The main assumption was that the first person was playing paired matches with the three other players. As in the previous datasets, we randomized the outcomes, assigning 1 for a win or 0 for a lost match. The two different types of rating scores calculations resulted in similar outcomes (see Fig. A1). The correlation between the two updated rating scores was .998, with small differences (*M* = 5.04, *SD* = 4.96, Min = 0.22, Max = 23.17). Taking everything into account, the results show that the two systems used in different datasets to calculate and update the rating scores produce rather similar patterns of rating scores, with very small differences between them.

## Footnotes

1 Tensor product functions are nonparametric, in the sense that we are not interested in the parameters, but rather in how well these smoothing parameters capture the behavior of a surface. In this way, we modeled wiggly surfaces resulting from this interaction; the reader is referred to Wood (2006). We used the restricted maximum likelihood option of the MGCV package (Wood, 2006) to obtain an optimal balance between undersmoothing and oversmoothing.
